# Designing Dynamic Hydrogels: The Interplay of Cross-Linker Length, Valency, and Reaction Kinetics in Hydrazone-Based Networks

**DOI:** 10.1021/acs.chemmater.4c02573

**Published:** 2025-04-02

**Authors:** Francis L. C. Morgan, Ivo A. O. Beeren, Lorenzo Moroni, Matthew B. Baker

**Affiliations:** † Department of Instructive Biomaterials Engineering, MERLN Institute for Technology-Inspired Regenerative Medicine, Maastricht University, 6229 ER Maastricht, The Netherlands; ‡ Department of Complex Tissue Regeneration, MERLN Institute for Technology-Inspired Regenerative Medicine, Maastricht University, 6229 ER Maastricht, The Netherlands

## Abstract

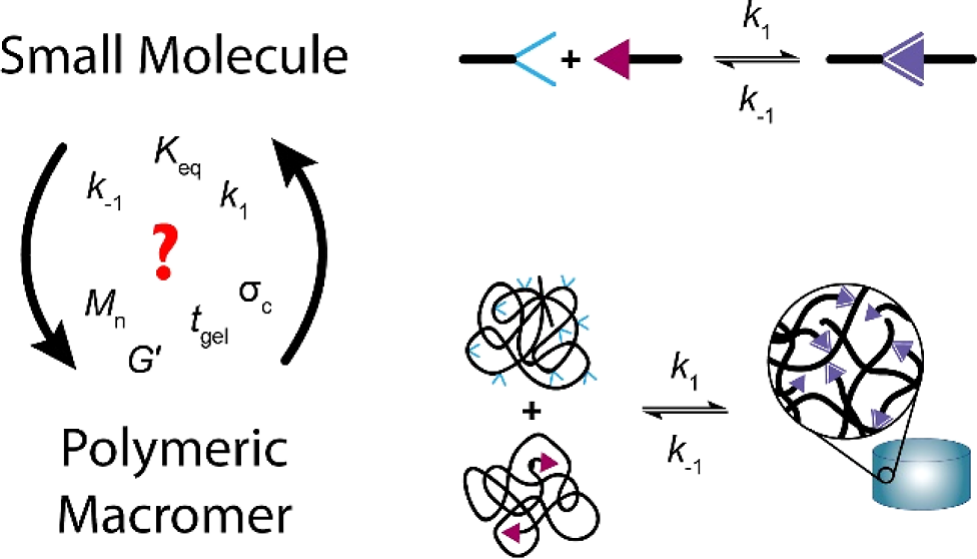

Hydrogels designed using dynamic (reversible) chemistry are prominent tools in diverse research areas as they grant access to time-dependent mechanical properties (self-healing and viscoelasticity), which are inaccessible via purely covalent networks. While the relationship between rate and equilibrium constants (RECs) and bulk mechanical properties is increasingly explored, less known is the effect of network topology or cross-linker length on both REC’s and mechanical properties in dynamically cross-linked hydrogels. Here, we chose hydrazone formation as a model system for dynamic covalent network formation. Using mono- and bivalent hydrazides with molecular weights of 0.1–20 kg·mol^–1^, we show that their chemical reactivity with a small molecule aldehyde is largely unaffected by their length. However, the apparent reactivity between two polymeric macromers revealed a decade reduction in *k*_1_ and *K*_eq_ compared with the model system. We then studied the impact of different cross-linkers on hydrogel mechanics, revealing a reduction in *G*′ of 1.3–2.5-fold (cross-linker length) vs 18–28-fold (cross-linker valency), along with emergent strain-stiffening behavior. Finally, we offer potential mechanisms for these observations. These results present a step forward for the rational design of dynamic hydrogel systems with targeted mechanical properties, particularly by facilitating the translation of model studies to practical (macromeric) applications.

## 1 Introduction

**Scheme 1:**
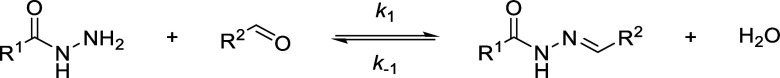
General Imine Formation Reaction between a Hydrazide and an Aldehyde

Hydrogels are a ubiquitous class of soft matter used prevalently in applications including tissue engineering,^1−5^ soft robotics,^6−8^ drug delivery,^9−12^ and printable (bio)electronics.^13,14^ Hydrogels can be formed in a variety of ways, including covalent bonding,^15−17^ physical interactions,^18−20^ and supramolecular association.^10,21−23^ More recently, dynamic covalent chemistry (DCvC)^24−27^ has emerged as a promising means of engineering desirable mechanical properties into hydrogels. DCvC is characterized by the reversible formation of a covalent bond and can be defined by the rate and equilibrium constants (RECs) for the formation (*k*_1_) and dissociation (*k*_–1_) of this bond (Scheme 1). Since the mechanical properties of hydrogels arise from the concentration and behavior of network junctions, hydrogels cross-linked via DCvC create an explicit relationship between the RECs and resulting mechanical properties.^28−31^ Leveraging this relationship has enabled specific mechanical regimes to be targeted,^32,33^ yet the rational design of these networks remains in its infancy. Notably, while the qualitative relationships among gelation kinetics (∝ *k*_1_), bulk stiffness (∝ *K*_eq_), flow (∝ *k*_–1_, *K*_eq_), and hydrogel persistence in vitro (∝ *k*_1_, *K*_eq_) are established, explicit determination of these constants is less common.

Among DCvC systems, hydrazone cross-linking is an established reaction for hydrogelation.^34,35^ Hydrazones are a type of imine formed by the reaction between a hydrazide and an aldehyde (Scheme 1), which are reversible (dynamic) under aqueous conditions: hydrazone formation is in equilibrium with imine hydrolysis. We recently studied the RECs for a series of dynamic imine reactions and found significant differences (1–2 decades) in the reactivity of model small molecule versus polymeric aldehydes.^36^ These differences highlight the need to determine experimental values of RECs in a hydrogel system and, more generally, to understand how these values in a practical system differ from model values. Understanding key differences between model and practical hydrogel systems is vital to advancing the bottom-up design of dynamic hydrogels.

A common approach to tuning hydrogel mechanics is via control of the length (molecular weight) of the cross-linker used. However, the effect of cross-linker length on the mechanical properties of dynamic hydrogels is not so clear. In contrast, the effect of cross-linker length on static covalent hydrogels has been explored by comparing the resulting elastic (*E*) or shear (*G*) moduli or other bulk mechanical properties (tensile strength, toughness, etc.).^37−41^ These structure–mechanics relationships in covalent materials are further codified in classical hydrogel models where differences in cross-linker length are typically a term in many phenomenological equations.^42,43^ These traditional approaches to covalent networks reveal a general trend of decreasing moduli and increasing strain-at-break (elasticity) with increasing molecular weight (length) when compared across the 1–20 kg·mol^–1^ range. Complicating the scenario further is the effect of the cross-linker length on the RECs for dynamic covalent reactions, which is also lacking. To address these gaps in DCvC knowledge and further assess how dynamic hydrogels differ from model systems, we begin by selecting both a model aldehyde and a copolymer macromer with pendant aldehyde groups. Then we investigate the RECs for the reaction of these aldehydes with homo- and bivalent hydrazides possessing tailing chain lengths ranging from 2 bonds to almost 1000 (see Design Of Kinetic Study to Investigate the Rate And Equilibrium Constants Of Hydrazone Formation). We then explore how the reactivity of different length cross-linkers translates to bulk properties through a rheological study of the resulting hydrogels. Additionally, we included tetravalent hydrazide cross-linkers to probe the relative impacts of RECs and network topology.

A deeper understanding of the impact of cross-linker length and valency on reactivity and mechanics is critical for the rational design of mechanically targeted hydrogels as well as for the comparison of diverse existing systems. Furthermore, cross-linker length and valency can be varied independently, thus offering additional opportunities when tuning hydrogel systems. While we performed this work using the common hydrazone linkage with two valencies at three length scales, we envision that our results could benefit the rational design of any dynamic cross-linking reaction and pave the way to develop future dynamic models.

## 2 Experimental Section

### 2.1 Materials

The synthetic copolymer with pendant aldehydes (**pSM-***co***-OMAm**, *M*_n_ = 36.4 kg·mol^–1^, aliphatic aldehyde in Table 1) was prepared as part of a previous work. For details regarding its preparation, see our previous publication.^44^

**Table 1:**
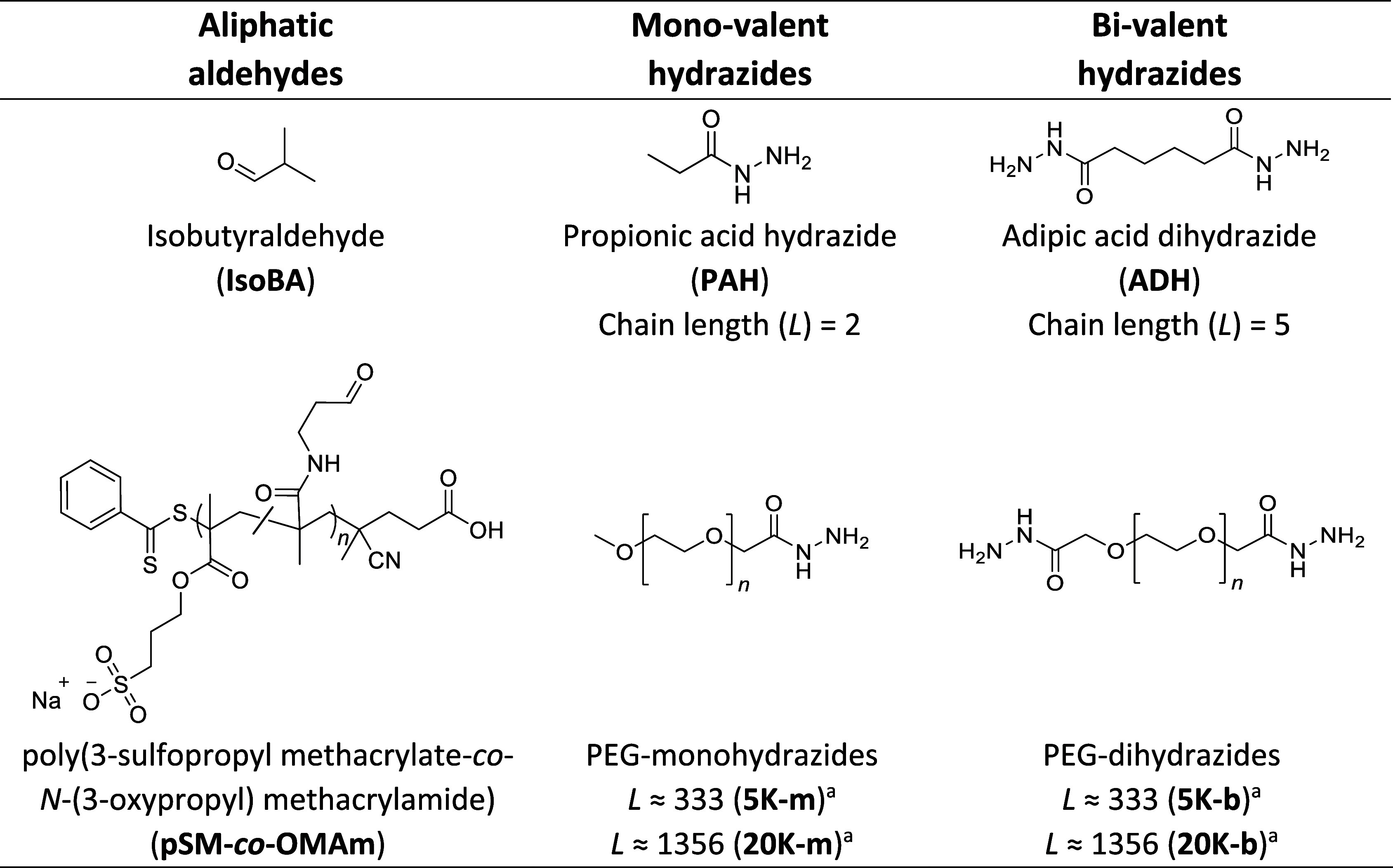
Aliphatic Aldehydes and Hydrazides Used in the Kinetic Study, along with the Chain Length (*L*) of Each Hydrazide

a The slight difference in chain length (*L*; from carbonyl to carbonyl/chain end) between mono- and bivalent hydrazides is neglected for ease of comparison as two bonds compared to a difference of several hundred is considered negligible. The values reported here are the midpoint between the values for the mono- and bivalent hydrazides.

Unless otherwise stated, all commercial materials were used as received. Bivalent 5 kg·mol^–1^ and 20 kg·mol^–1^ 2-arm polyethylene glycol (PEG) hydrazides (**5K-b** and **20K-b**) were purchased from BiopharmaPEG (HO019019-5K, HO019019-20K; *D̵* ≤ 1.05) while tetravalent 5 kg·mol^–1^ and 20 kg·mol^–1^ 4-arm PEG hydrazides (**5K-t** and **20K-t**) were purchased from Creative PEGWorks (PSB-4067, PSB-4069; *D̵* = 1.02–1.05). Adipic acid dihydrazide (**ADH**) (A0638-25G) and isobutyraldehyde (**IsoBA)** (240788-2 ML) were purchased from Sigma-Aldrich. Finally, Gibco Phosphate Buffered Saline (pH 7.4, without calcium or magnesium, 10010056) was purchased from Fisher Scientific.

### 2.2 UV–Vis Kinetic Measurements

**Figure 1:**
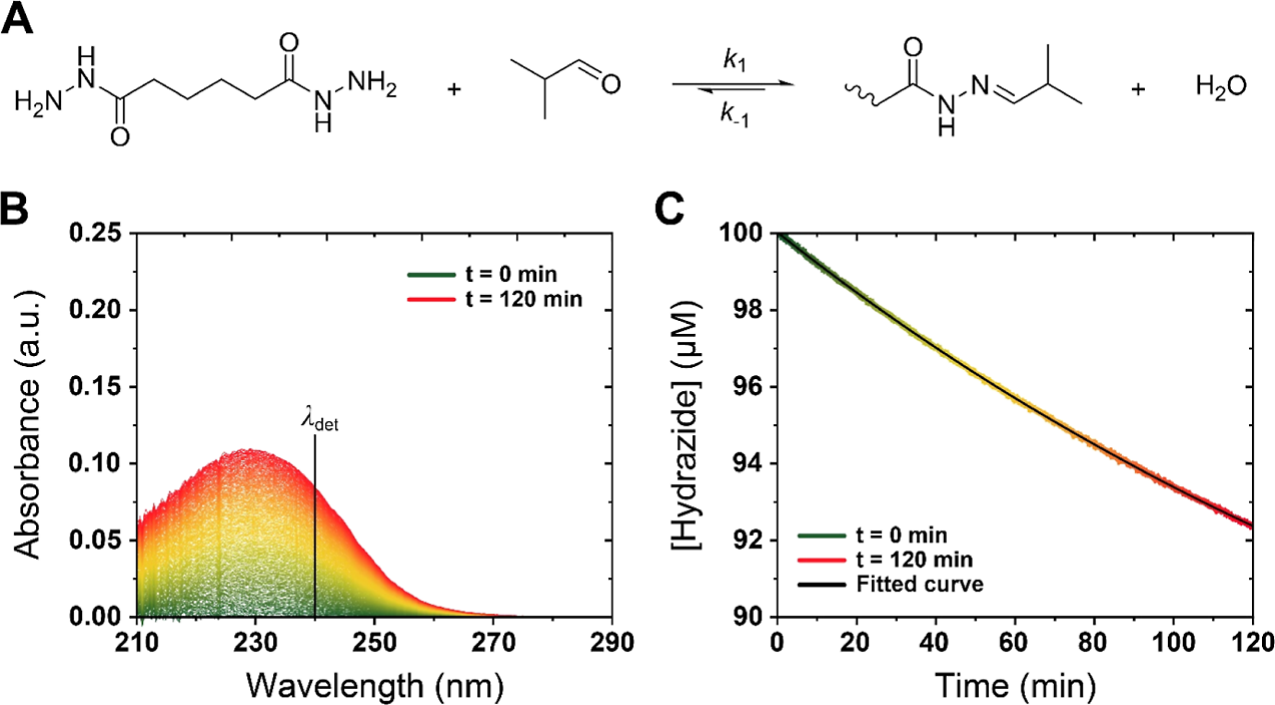
Example UV–vis data acquisition for adipic acid dihydrazide (**ADH**) and isobutyraldehyde (**IsoBA**). (A) Example reaction between **ADH** and **IsoBA**. (B) Typical UV–vis traces for a 2 h **IsoBA-ADH** kinetic measurement showing the change in absorbance for hydrazone formation over time. (C) Example of a processed **IsoBA**-**ADH** replicate with fitting (black lines) of these data to a reversible bimolecular kinetic model (see Experimental Section and Figure S3) to extract *k*_1_ and *k*_–1_. *K*_eq_ is calculated as *k*_1_/*k*_–1_. The detection wavelength (λ_det_) for hydrazone is taken at 240 nm with ε = 11,100 L·mol^–1^·cm^–1^ and 8600 L·mol^–1^·cm^–1^ for hydrazone formation with isobutyraldehyde and **pSM-***co***-OMAm**, respectively.^36^

UV–vis spectroscopic measurements were performed on an Agilent Cary 60 UV–vis spectrophotometer equipped with a Peltier temperature controller. Spectra were acquired every 30 s for 7200 s (2 h) from 300 to 200 nm with a scan rate of 396 nm·min^–1^ and steps of 0.33 nm at 20 °C. For each measurement, a quartz cuvette (Hellma Analytics, 114F-10-40, light path = 10 mm × 4 mm) was first cleaned with water and ethanol and then dried with compressed air. The absorbance signal was zeroed against air, and a baseline of PBS was recorded. The aldehyde (**pSM-***co***-OMAm** or **IsoBA**) was added from 10 mM (aldehyde functions) stock in PBS and thoroughly mixed by pipetting 200 μL up and down ≈25 times. The sample was allowed to equilibrate thermally for 10 min prior to the addition of the hydrazide from 20 mM (hydrazide functions) stock in PBS. The now reacting mixture was homogenized by pipetting, and data acquisition was started exactly 25 s after hydrazide addition. Kinetic data (Figures 1, S1, and S2) are reported from 210 nm as this corresponds to the solvent cutoff wavelength for PBS.

### 2.3 Fitting of UV–Vis Data to a Second-Order Bimolecular Reversible Rate Equation to Obtain Rate and Equilibrium Constants

Kinetic data were processed by subtracting the first spectrum from all other spectra to correct for the background signal from hydrazide and aldehyde reagents prior to imine formation. We thus obtain the change in absorbance over time, which is converted (using ε) to a plot of concentration over time for the reacting amine (not imine) (see Figure S3). The forward (*k*_1_) and reverse (*k*_–1_) rate constants for hydrazone formation were determined by fitting the kinetic data to the solution for an equimolar, bimolecular, reversible reaction following second-order kinetics developed by Dirksen et al.—reproduced below for convenience.^45^ The detection wavelength (λ_det_) for hydrazone is taken at 240 nm with ε = 11,100 L·mol^–1^·cm^–1^ and 8600 L·mol^–1^·cm^–1^ for hydrazone formation with **IsoBA** and **pSM-***co***-OMAm**, respectively, as previously determined.^36^Eq 1 describes the disappearance of the hydrazide in time, where *x*_0_ is the initial hydrazide concentration and *x*(*t*) is the remaining hydrazide concentration as some point in time (*t*). Fitting was done by programming eq 1 into Origin 2018 (OriginLab).
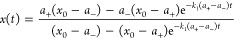
1where
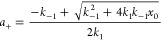
and
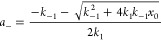


### 2.4 Hydrogel Preparation

Stock solutions were prepared in PBS of the synthetic copolymer with pendant aldehydes (**pSM-***co***-OMAm**, 49.3 mM aldehydes), alongside each of the hydrazide cross-linkers used in this work (**5K-b** at 47.6 mM; **5K-t** at 44.9 mM; **20K-b** at 52.0 mM; **20K-t** at 44.3 mM; concentrations are of hydrazide functions). More details can be found in Table S1. If necessary, stock solutions were neutralized to pH 7.4 (**5K-b** and **20K-b** were slightly acidic after dissolution). Hydrogels were prepared by first mixing the required amount of PBS to achieve the correct final concentrations (see Table 4) to the necessary volume of the hydrazide stock solution (as these were typically more viscous). Then the aldehyde stock solution and hydrazide solution were mixed as needed to form a hydrogel.

### 2.5 Rheological Analysis of Hydrogels

Rheological measurements were performed on a DHR-2 rheometer from TA Instruments, equipped with a solvent trap and with Peltier temperature-controlled bottom geometry a 20 mm cone–plate upper geometry. Samples were prepared according to the compositions in Table 4 with a final volume of 95 μL: 84 μL to load into the rheometer and 11 μL excess to allow for traces in the Eppendorf tube.

First, the hydrazide stock was mixed with the PBS necessary to achieve the correct final concentrations. The **pSM-***co***-OMAm** stock was then carefully added to the side of the Eppendorf (not yet mixing with the hydrazide solution). Once the Eppendorf was sealed, rapid inversion and 2–3 s of vortexing allowed the fastest loading of the sample onto the rheometer.

**Figure 2:**
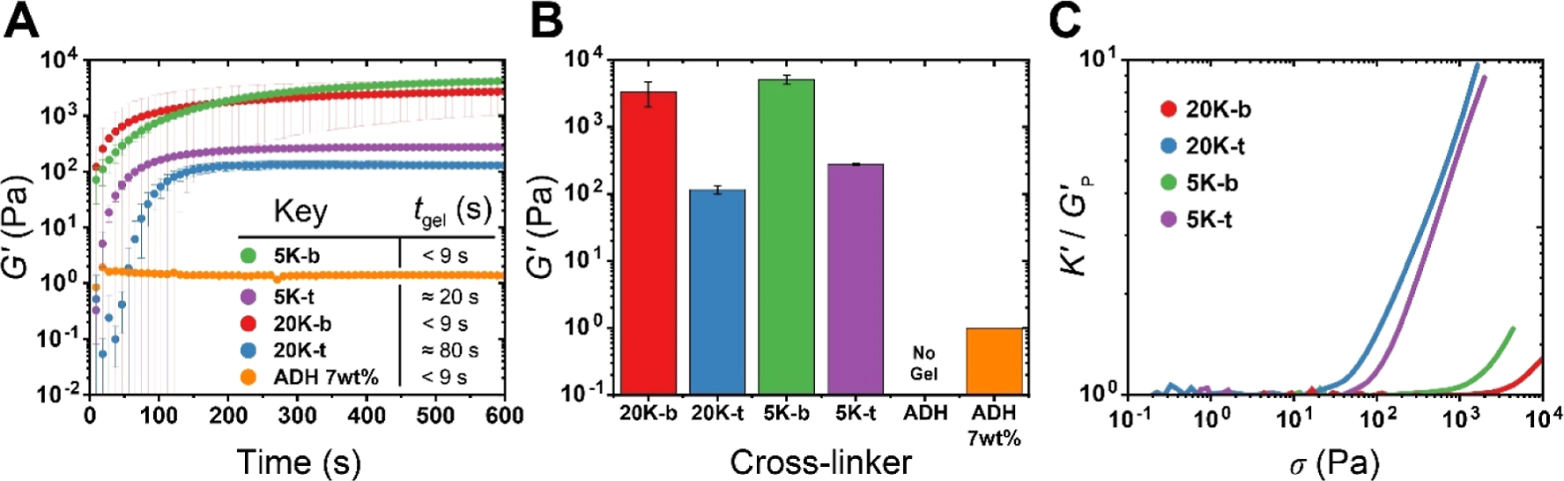
Rheological study of bi- and tetravalent PEG hydrazides forming gels with **pSM**-*co*-**OMAm** copolymer at a constant equimolar aldehyde and hydrazide concentration in PBS, at pH = 7.4 and 20 °C. (A) Time sweeps (γ = 1%, ω = 1 rad·s^–1^, *t* = 1800 s, *T* = 20 °C) performed to monitor cross-linking kinetics over time; *G*″ is omitted from the main figure for visual clarity (see Figure S4). The inset provides the key and associated hydrogelation time (*t*_gel_)—defined here as onset time of a rapid increase in *G*′. (B) Final shear moduli (*G*′) attained after 1800 s for the same formulations. Values given are the mean ± standard deviation for 2–3 replicates, with the exception of ADH 7 wt %, which is a single measurement. Time sweep covering the full 1800 s along with frequency sweeps can be found in Figure S4. (C) Representative normalized differential modulus (*K*′ = ∂σ/∂γ) highlighting the strain-stiffening behavior of these formulations. Raw strain sweeps and replicates for *K*′ can be found in Figure S5.

In the case of **20K-b**, a different strategy was needed as cross-linking proceeded too fast to successfully load the sample into the rheometer. First, the hydrazide solution was loaded onto the bottom geometry of the rheometer, while the **pSM-***co***-OMAm** solution was pipetted onto the underside of the upper geometry, where it was held by surface tension. Then, the upper geometry was lowered while rotating; the shear induced by the rotation was used to mix the solutions rapidly while reaching the measurement gap. This did allow us to acquire the final stages of cross-linking, but the sample loading remains relatively heterogeneous and led to quite a large variation (Figure 2A).

Time sweeps were recorded at 1% strain and 1 rad·s^–1^ for 1800 s at 20 °C. This was followed by a frequency sweep from 1 to 100 rad·s^–1^ at 1% strain and 20 °C and finally a strain sweep from 0.1 to 1000% at 1 rad·s^–1^ and 20 °C. Final shear moduli values were taken as the average value of the plateau moduli during frequency sweeps. The differential modulus (*K*′ = ∂σ/∂γ) was determined from the strain sweeps by taking the derivative of the oscillation stress w.r.t. strain. This stiffening index, *m*, was given by the slope to a linear fit of log(*K*′) vs log(σ) (as *K*′ ∝ σ^*m*^) for the final 5 points on the stiffening curve prior to rupture. Similarly, the critical strain (σ_c_) was determined from the intersection of the same linear fit with the plateau modulus.

### 2.6 Dynamic Light Scattering Measurements of Dilute Solutions

Stock solutions in PBS of **pSM-***co***-OMAm** (2 mg·mL^–1^, ≈2.5 mM aldehyde), **20K-b** (32 mg in 203 μL; ≈15 mM hydrazide), and **20K-t** (34.5 mg in 219 μL; ≈30 mM hydrazide) were prepared. Mixtures composed of 525 μL of **pSM-***co***-OMAm**, with either 42.5 μL of **20K-t** or 85.0 μL of **20K-b** were then made up to 1000 μL with PBS giving a final density adjusted concentration of **pSM-***co***-OMAm** of 1 mg·mL^–1^ with equimolar concentrations of aldehyde and hydrazide functions. These dilute solutions were allowed to react for 2 h prior to the acquisition of dynamic light scattering (DLS) data. DLS measurements were performed using a Malvern Zetasizer Nano ZSP instrument in a disposable plastic cuvette containing 1000 μL of sample solution and analyzed with the accompanying Zetasizer software. Note that the apparent particle sizes shown in Figure S6 should not be considered absolute but only relative to one another as the conditions (spherical, known absorbance, and refractive index) assumed by the software have not been verified.

## 3 Results and Discussion

### 3.1 Design of Kinetic Study to Investigate the Rate and Equilibrium Constants of Hydrazone Formation

To investigate the effect of cross-linker length on RECs, we first selected aliphatic, mono-, and bivalent hydrazides. Beginning with small model molecules, we chose propionic acid hydrazide (**PAH**) and adipic acid dihydrazide (**ADH**) as model studies. For comparing cross-linker length, we included PEG hydrazides with molecular weights of either 5 kg·mol^–1^ (**5K-m**, **5K-b**) or 20 kg·mol^–1^ (**20K-m**, **20K-b**), representative of commonly used lengths of PEG-based cross-linkers in the literature. The notation *X*K-m/b/t refers to PEG of *X* kg·mol^–1^, with the m/b/t suffix classifying them as mono- (m), bi- (b), or tetra- (t) valent. Monovalent homologues of each hydrazide size are included to allow studies of their reactivity with aldehyde macromers while avoiding cross-linking during kinetic UV–vis measurements; in situ cross-linking may change the scattering background over time as larger aggregates form. To facilitate the comparison of the cross-linker size, we define the chain length (*L*) as the number of bonds between a carbonyl carbon of a hydrazide group and either the carbonyl carbon or methyl at the opposite end of the chain, which varies from 2 to 1356 (Table 1).

Two aldehydes were then selected to react with these hydrazides. We chose **IsoBA** as a model small molecule, as well as the same copolymer macromer with pendant aldehyde groups (**pSM-***co***-OMAm**) that we used in our previous work on determining RECs of imine formation.^36^ Studying both aldehydes independently allows the comparison of linker lengths as well as comparison between model small molecule studies and macromers used in hydrogel formation. The structures, abbreviations, and chain lengths of the aldehydes and hydrazides used in this kinetic study are summarized in Table 1.

RECs for pairs of amines and aldehydes were determined by UV–vis spectroscopy as previously described using the integrated solution to a second-order equimolar bimolecular reversible rate equation (See eq 1 in the Experimental Section).^36,45^ In our previous work, we tested this fitting method using kinetic data obtained via NMR and UV–vis, and compared *k*_1_ values to a pseudo-first-order kinetic fit. However, we do note that the *k*_–1_ values obtained via this method may exhibit some variation as they become many orders of magnitude smaller than the accompanying *k*_1_ values; in future work, we aim to make comparisons with additional methods such as isothermal titration calorimetry to further validate the accuracy of this approach as a general analytical method for the quantification of RECs in dynamic chemical systems. An example UV–vis measurement and subsequent fitting for the reaction between **ADH** and **IsoBA** are given in Figure 1 to illustrate data acquisition and processing, while the remaining kinetic data can be found in Figures S1 and S2.

### 3.2 Effect of Hydrazide Cross-Linker Length (Molecular Weight) on Rate and Equilibrium Constants When Reacting with a Model Aldehyde

We first began by studying the effect of cross-linker length on RECs by reacting bivalent hydrazides with **IsoBA**, our model aldehyde. We also chose to include data from **PAH** + **IsoBA** as a reference from our previous work to facilitate comparison and provide a bridge for comparing mono- and bivalent hydrazides.^36^ As summarized in Table 2, when we increased the hydrazide length from *L* = 5 (**ADH**) to *L* ≈ 1356 (**20K-b**), we observed no significant impact on the apparent forward rate constant (*k*_1_). However, the reverse rate constant of both PEG cross-linkers (**5K-b** & **20K-b**) was increased 2–3 fold, resulting in lower equilibrium constants. This small increase in *k*_–1_ may be due to the β-oxygen atom present in PEG hydrazides compared to carbon chains in **PAH** and **ADH**, though a separate investigation would be needed to determine if such electronic effects underlie this observation. No meaningful difference was observed between the mono- (**PAH**) and bivalent (**ADH**) small-molecule hydrazides, which is unsurprising given their similar size and electronic structure. Overall, the RECs of the hydrazides that reacted with **IsoBA** showed low sensitivity to chain length as well as to the presence of the β-oxygen atom present in PEG chains, with at most a 3-fold difference in *k*_–1_ and the corresponding *K*_eq_.

**Table 2:** RECs Obtained from the Model Kinetic Study of **PAH** and Different Bivalent Hydrazide Cross-Linkers Reacting with **IsoBA**^b^

hydrazide	aldehyde	MW_hydrazide_ (g·mol^–1^)	*k*_1_ (10^–1^·L·mol^–1^·s^–1^)	*k*_–1_ (10^–5^·s^–1^)	*K*_eq_ (10^3^·L·mol^–1^)
**PAH**	**IsoBA**	88.1	1.2 ± 0.0(3)^a^	5.7 ± 0.1^a^	2.2 ± 0.0(8)^a^
**ADH**	**IsoBA**	174.2	1.3 ± 0.0(3)	4.5 ± 0.4	3.0 ± 0.2
**5K-b**	**IsoBA**	5000	1.4 ± 0.1	13.2 ± 0.7	1.1 ± 0.0(3)
**20K-b**	**IsoBA**	20,000	1.1 ± 0.1	9.3 ± 0.7	1.2 ± 0.0(2)

a Values taken from our previous work.^36^

b Values reported are the mean ± standard deviation of 3–4 replicates.

### 3.3 Comparison of Small Hydrazide Reactivity with a Model Aldehyde vs an Aldehyde Macromer

Next, we investigated the reactivity of **PAH** with our aldehyde macromer, **pSM-***co***-OMAm** (Table 3, data for **PAH** + **pSM-***co***-OMAm** taken from our previous work^36^). We observed that **pSM-***co***-OMAm** is more reactive than **IsoBA**, with a 5.7-fold increase in *k*_1_ (6.9 × 10^–1^ L·mol^–1^·s^–1^ vs 1.2 × 10^–1^ L·mol^–1^·s^–1^) and a concomitant increase in *K*_eq_.^36^ This result indicated that our synthetic aldehyde macromer is more reactive than **IsoBA**. We previously hypothesized that this noticeable increase in forward rate constant could be due to the stabilization of the tetrahedral intermediate via an H-bonded 8-membered ring.^36^ This proposal is consistent with similar intermediate trapping or transition-state stabilization arguments that have been proposed to explain the relative reaction rates in a series of aromatic hydrazones.^34,46,47^

**Table 3:** RECs Obtained from the Model Kinetic Study of Different Monovalent Hydrazides Reacting with an Aldehyde Containing Macromer (**pSM**-*co*-**OMAm**)^b^

hydrazide	aldehyde	MW_hydrazide_ (g·mol^–1^)	*k*_1_ (10^–1^·L·mol^–1^·s^–1^)	*k*_–1_ (10^–5^·s^–1^)	*K*_eq_ (10^3^·L·mol^–1^)
**PAH**	**pSM**-*co*-**OMAm**	88.1	6.9 ± 0.3^a^	5.7 ± 0.3^a^	12.0 ± 0.7^a^
**5K-m**	**pSM**-*co*-**OMAm**	5000	0.46 ± 0.05	3.4 ± 0.8	1.2 ± 0.2
**20K-m**	**pSM**-*co*-**OMAm**	20,000	0.75 ± 0.05	7.8 ± 3.3	0.9 ± 0.3

a Values taken from our previous work.^36^

b Values reported are the mean ± standard deviation of 3–5 replicates.

### 3.4 Effect of Hydrazide Cross-Linker Length (Molecular Weight) on Rate and Equilibrium Constants When Reacting with a Polymeric Aldehyde Macromer

We were then interested in moving to the RECs between two macromers, as encountered in dynamic hydrogel formation. This is important because many reported systems use a macromer with pendant functional groups as part of their dynamic covalent network formation, as opposed to exclusively using small molecules or telechelic PEG macromers. Consequently, understanding the differences in dynamic covalent reactivity that can be expected between model studies and employed macromers is an important design consideration. To investigate this, we used monovalent hydrazides to avoid cross-linking the **pSM-***co***-OMAm** during the UV–vis kinetic data acquisition. The obtained RECs between monovalent hydrazides and **pSM-***co***-OMAm** are summarized in Table 3.

Compared to **PAH** + **pSM-***co***-OMAm**, the reaction between PEG hydrazides and **pSM-***co***-OMAm** yielded very similar *k*_–1_ values, but *k*_1_ values were 9–15-fold lower and resulted in *K*_eq_’s 1 order of magnitude lower (≈1.0 × 10^3^ L·mol^–1^ vs 1.2 × 10^4^ L·mol^–1^). Notably, in our recent work,^36^ we showed that the range of *K*_eq_’s that most strongly affects the reacted fraction of imines (when reactive group concentrations ≈10^1^ mM) falls between 10^2^ L·mol^–1^ and 10^4^ L·mol^–1^. Thus, shifting equilibrium constants into this regime highlights how the effective reactivity of macromers compared to small molecules can significantly impact a system designed from the bottom-up. We hypothesize that this observed drop in *k*_1_ for a pair of macromers is likely due to the kinetic excluded volume effect (resistance to chain interpenetration) of the two macromers—**pSM-***co***-OMAm** has an *M*_n_ of 36.4 kg·mol^–1^ compared to a molar mass of 72 g·mol^–1^ for **IsoBA**.^48,49^ Morawetz performed seminal work modeling the excluded volume effect on reaction in rates in polymeric versus small-molecule model systems and predicted a decrease in the relative forward rate constant that scales with the logarithm (log*k*/*k*_0_ ∝ log*x*) of the number of carbon–carbon bonds (*x*)—analogous to *L* in this work. Black and Worsfold later compared some of these models to experimental data derived from both models, polymeric and mixed (one polymeric macromer and one model molecule) systems, and found a 3–24 fold decrease in the apparent rate when both reactants were polymeric versus a mixed system.^50^ These data are consistent in magnitude with our observed 10-fold decrease, although we also note that the magnitude of the decrease reported by Morawetz was dependent on the concentration of the polymeric species as well as the chain length. The latter scaling with log(*x*) may explain why we see little difference between **5K-m** (*L* ≈ 333) and **20K-m** (*L* ≈ 1356) compared to **PAH** (*L* = 2). A more recent study explored this behavior in synthetic organic polymeric systems and found that the excluded volume expansion factor was near unity for short chains (i.e., there is no change in occupied volume) and crossed over to a power law dependence on chain length for longer chains (solvated chains occupy a larger relative volume as their length increases).^51^ These results reinforce the importance not only of macromolecular properties (e.g., molecular weight, degree of functionalization) on effective reaction rates but also of macromer conformation, solvation, and excluded volume in precursor solutions.

### 3.5 Rheological Investigation of Hydrogel Cross-Linking Rates Using Hydrazides of Different Lengths and Valency

With RECs for our hydrazide and aldehyde pairs determined, the next step was to explore how they translate to macroscopic hydrogel properties using our bivalent hydrazide cross-linkers **pSM-***co***-OMAm**. For this investigation, we additionally included tetravalent PEG hydrazide variants of our cross-linkers to assess the relative impact of cross-linker valency—another common design parameter in published systems.^40,52−55^

We chose to keep the concentration of reactive groups constant and equivalent across all formulations ([aldehyde] = [hydrazide] = 18.8 mM) so that the maximum number of chemical cross-links that may be formed remains constant. However, given the large difference in molecular weight of bivalent PEG hydrazides (5K vs 20K) compared to **ADH**, the total mass content of each hydrogel varies. Similarly, the tetravalent variants will contain half as many cross-linker molecules to maintain a constant hydrazide concentration; furthermore, we must acknowledge that the branch lengths between the bi- and tetravalent cross-linkers are also changing in our experimental setup. The formulations and associated mass content are described in Table 4.

**Table 4:** Composition of Hydrogels Prepared for the Rheological Study of Hydrogel Mechanics as a Function of Cross-Linker Length and Valency

[**pSM**-*co*-**OMAm**] (wt %)	[aldehyde] (mM)	equiv of hydrazide	cross-linker	total solids (wt %)
1.5	18.8	1	**20K-b**	21.3
1.5	18.8	1	**20K-t**	11.4
1.5	18.8	1	**5K-b**	6.5
1.5	18.8	1	**5K-t**	4.0
1.5	18.8	1	**ADH**	1.7
7.0	89.4	1	**ADH**	7.8

Looking first at the cross-linking kinetics of **pSM-***co***-OMAm** with each of the bivalent hydrazides, we were initially surprised when ADH failed to form a gel. We attribute this to intramolecular cross-linking dominating the reaction as opposed to intermolecular cross-linking, which is discussed in more detail later. In contrast, we have previously reported hydrogels (2 wt %, [aldehyde] = 10 mM) composed of **ADH** and oxidized alginate (10% oxidized uronic acid units with *M*_n_ = 130 kg·mol^–1^).^32^ The inability of **pSM-***co***-OMAm** (29% aldehyde containing units with *M*_n_ = 36.4 kg·mol^–1^) to form a hydrogel with **ADH** highlights how the molecular weight between cross-links, as well as overall chain and persistence () length, affects hydrogelation. Indeed, increasing the **pSM-***co***-OMAm** content to 7 wt % while retaining 1 equiv of **ADH**—almost a 5-fold increase in both polymer content and cross-linker concentration—only led to the formation of a very weak (*G*′ = 1 Pa) gel (Figure 2). However, this hydrogel was almost completely cross-linked before acquisition on the rheometer could be started (<9 s), indicating that network formation was extremely rapid. Here, we define the cross-linking time as the onset of a rapid increase in the level of *G*′. Full time sweeps covering 1800 s can be found in Figure S4.

Considering next the relative cross-linking times using the bivalent PEG hydrazide cross-linkers, we observed rapid (<90 s) cross-linking in all formulations. Comparing 5K hydrazides to 20K hydrazides, we observed that the longer cross-linkers had no clear impact on the rate of bivalent cross-linking, while for the tetravalent linkers, the shorter **5K-t** cross-linked slightly faster than **20K-t** (Figure 2A).

Interestingly, both **5K-t** and **20K-t** began cross-linking slower than their bivalent counterparts did; however, both tetravalent cross-linkers attained a stable plateau modulus (thermodynamic equilibrium) much more rapidly than the bivalent hydrazides (<200 s compared to >600 s) (Figure 2A). This observation is quite intriguing as it does not clearly align with reasoning based on common parameters such as mass content and chain diffusion nor reactivity (*k*_1_). If high mass content limited chain diffusion, we would expect **5K-b** (6.5 wt %) to reach a plateau faster than **20K-t** (11.4 wt %), but we do not observe this. We explicitly measured the relative reactivity of each PEG hydrazide (Tables 2 and 3) and found no significant differences; these findings preclude reactivity from being the cause of this observation. We believe that an increase in intramolecular cross-linking may also explain this behavior, which we discuss in more detail later. While the curing time (traditionally for the kinetic product of irreversible and nondynamic processes such as radical reactions) has been well described, the time needed for dynamic hydrogels to reach their plateau modulus (thermodynamic equilibrium) lacks modern models to describe our observations. Though undoubtedly interesting, and a target for future developments, an investigation into this theory is beyond the scope of the current work.

### 3.6 Rheological Investigation of Hydrogel Mechanical Properties Using Hydrazides of Different Lengths and Valency

Moving on to the final *G′* obtained for each formulation (Figure 2B), we also observed an interesting difference; the shorter cross-linkers resulted in higher shear moduli (**5K-b** ≈ 5100 Pa > **20K-b** ≈ 3400 Pa, and **5K-t** ≈ 280 Pa > **20K-t** ≈ 120 Pa), despite having significantly lower solids content. This relative drop in shear moduli can be rationalized in the framework of conventional covalent network theory, using the approximate relationship described below by eq 2 (assuming an affine network).^56,57^
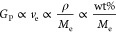
2where *G*_P_ is the plateau modulus (stable *G*′ after network formation), ν_e_ is the density of entanglement (elastically active) strands, ρ is the polymer density, related here to the mass content of each formulation, and *M*_e_ is the molecular weight between elastically active entanglements. Indeed, this simplified estimation of *G*_P_ proportionality using the known values for wt %, and approximating *M*_e_ as the ideal molecular weight between PEG functional groups (e.g., *M*_e_ of **20K-t** = 20*K*/2) for each bi- or tetra-valent pair (**5K-b** vs **20K-b**, and **5K-t** vs **20K-t**) yields expected relative decreases of 18% and 29%, respectively, which fall within the measurement uncertainty.

However, comparing the bi- and tetravalent cross-linkers provides an unexpected result. The shear storage moduli obtained using tetravalent cross-linkers are over an order of magnitude lower than those for bivalent cross-linkers—5100 Pa (**20K-b**) and 3400 Pa (**5K-b**) compared to 280 Pa (**20K-t**) and 120 Pa (**5K-t**). This dramatic difference does not follow the total mass content of the formulation nor the approximate relationship described above. Typically, an increase in the mass content for a fixed chemical cross-link concentration leads to a proportional increase in stiffness. We observe this in the case of varying cross-linker length but not for varying cross-linker valency. We propose an explanation for these observations in the next section.

Apart from their moduli, the frequency and strain behavior of formed hydrogels are often characterized to provide insight into their viscoelasticity (time-dependent mechanics) and strain-stiffening (important for biomimicry and processing, for example). Following in situ cross-linking, we measured the frequency-dependent behavior of our cross-linked hydrogels. Both bivalent formulations showed frequency-independent behavior characteristic of the plateau region across the measured range (1–100 rad·s^–1^, Figure S4). In contrast, the softer tetravalent formulations, while predominantly frequency-independent across the same range, exhibit an increase in *G*″ and decrease in *G*′ as frequency decreases, forecasting a crossover point (λ) below 1 rad·s^–1^. This crossover point also enables the approximation of the characteristic stress relaxation time (λ ≈ 1/τ), which is relevant for many applications.^58,59^

Finally, we also investigated the nonlinear viscoelastic response of our hydrogels with oscillatory amplitude sweeps and found them all to possess strain-stiffening behavior (Figures 2C and S5). This is notable as there are few examples of purely synthetic systems that possess this property.^60−64^ Strain-stiffening is common in biological systems, so synthetic materials that can control and mimic this behavior are highly sought after. We recently showed that **pSM-***co***-OMAm** hydrogels with a varying fraction of aldehydes exhibited a decrease in critical strain (σ_c_, onset of strain stiffening) and a slight increase in stiffening index (*m*; a measure of the magnitude of the stiffening response) with decreasing polymer concentration. The decrease in total mass content was accompanied by a concomitant decrease in stiffness.^44^ Interestingly, in this study, our bivalent formulations show similarly high σ_c_ (≈1000 Pa) and low *m* (≈0.17, Table S1, See Experimental Section) values, despite a significant difference in mass content (**5K-b** = 6.5%; **20k**-**b** = 21.3%). Notably, our previous study varied the aldehyde fraction and/or mass content of **pSM-***co***-OMAm** (and thus total aldehydes), while the current study maintains a constant functional group concentration, with mass differences coming from the length of PEG chains.

If we further compare these values to their tetravalent counterparts, we observed a large decrease in σ_c_ (≈100 Pa) and a concomitant increase in *m* (≈0.74) irrespective of the differences in the mass content. These results highlight the potential importance of network topology for tuning strain-stiffening and highlight increased cross-linker valency as a potential means to reduce the critical strain for targeting the biological regime. The ability to tune the onset and magnitude of strain-stiffening behavior is necessary for mechanically matching the stiffening response of hydrogels to a chosen application and is particularly powerful when control can be achieved independently of cross-link concentration and chemistry as we see in our system. Though to fully take advantage of these behavioral trends, future work to systematically vary both linker length and linker valency independently across a wider range will be necessary.

Overall, the length of PEG hydrazide cross-linkers had only a small impact on their RECs, and resulting hydrogel stiffness and cross-linking kinetics. The appearance of strain-stiffening behavior positions these dynamic hydrogels as promising candidates for future biomedical applications. However, more importantly, this study identifies cross-linker valency as a more potent method for tuning hydrogel stiffness, cross-linking behavior, and critical strain independently of the RECs that govern dynamic networks. While our results clearly demonstrate the importance of considering network topology in conjunction with the cross-linker length and reactivity when designing dynamic systems, traditional reasoning based on mass content and RECs is insufficient to explain all of our observations fully. In the next section, we propose possible mechanisms to rationalize our observations and identify avenues of future exploration.

### 3.7 Proposed Mechanisms to Describe the Observed Trends in Hydrogel Behavior

Throughout our discussion, we described several observations that did not immediately follow the trends we would expect based on conventional reasoning of network mechanics with respect to mass content and cross-linker valency. In the present discussion, we offer possible explanations for the unexpectedly large difference in stiffness, time to plateau modulus, and strain-stiffening that tetravalent hydrazide cross-linkers induced in our hydrogels compared to their bivalent counterparts, as well as the inability of **pSM-***co***-OMAm** to gel at 1.5 wt % with a small-molecule cross-linker (**ADH**).

At a fundamental level, the mechanical response of a hydrogel is governed by both chain properties (chain rigidity, persistence length, chain length, solvent interaction, radius of gyration, etc.) and network properties (chain density, junction density, and type—e.g., physical entanglement versus chemical cross-link, as well as the functionality of each cross-linking node). While the chain properties are inherent to the chosen polymers, the network properties are also related to reaction parameters for a given cross-linking reaction (*k*_1_, *k*_–1_, *K*_eq_, chain mobility/diffusion, molar ratio of reactive groups, and accessibility of reaction groups). For a given hydrogel system, many of these reaction parameters remain constant, enabling a more straightforward analysis of the relationship between chain and network properties with bulk mechanics.

**Figure 3:**
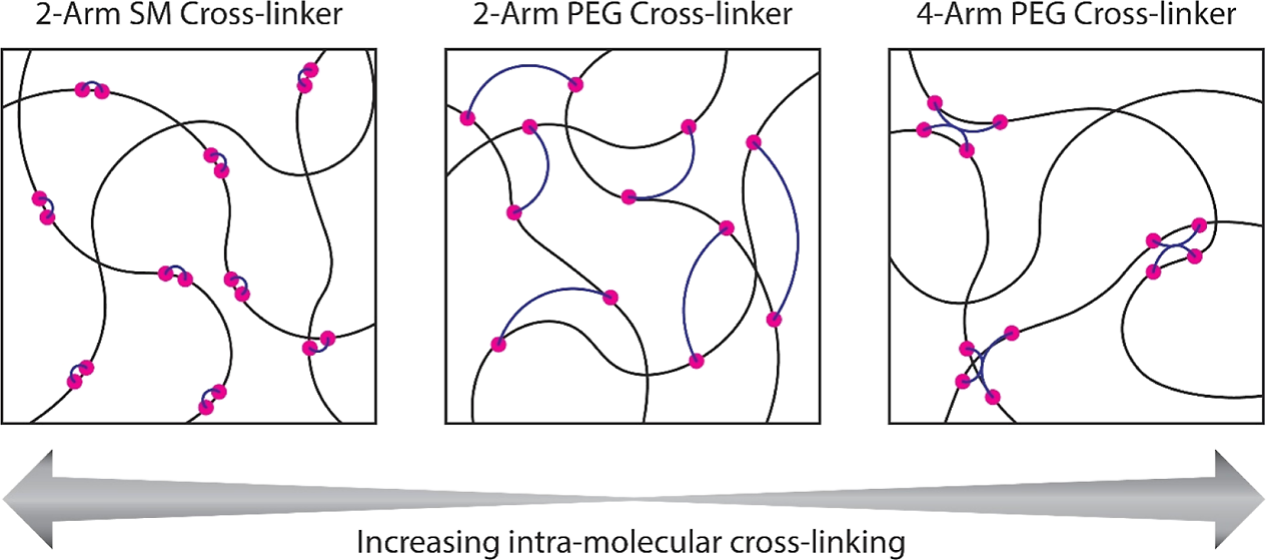
Schematic of the proposed differences in inter- vs intramolecular cross-linking for different cross-linking lengths and valencies. (Left) A small-molecule (SM) cross-linker such as the **ADH** used in this work will primarily bind an adjacent functional group on a highly functionalized macromer, favoring intramolecular cross-linking. (Middle) A bifunctional 2-arm PEG hydrazide cross-linker will primarily bind different macromers given the spacing between the reactive groups and excluded volume effects. (Right) A tetrafunctional 4-arm PEG hydrazide cross-linker of an equivalent molecular weight will favor an increased fraction of intramolecular cross-linking due to the higher effective local concentration of connected hydrazides [arising from a shorter mean-square radius of gyration (*R*_g_) and higher valency].

Considering first the inability of **pSM-***co***-OMAm** to gel at 1.5 wt % with **ADH**, this can be reasoned based on the density of reactive aldehyde groups on the **pSM-***co***-OMAm** backbone (29%). The high local density of aldehydes relative to the chain density in solution (1.5 wt %), in conjunction with the small size of **ADH** (precluding excluded volume effects), provides a statistical argument for a predominance of intra- instead of intermolecular cross-linking (Figure 3). Once one end of **ADH** binds to a free aldehyde, it is much more likely to encounter an aldehyde on the same polymer chain as opposed to a different polymer chain.^65−67^ Overcoming this effect would require a polymer concentration well into the semidilute regime such that the degree of chain overlap favors intermolecular cross-linking over intramolecular cross-linking, which may be approached as we increase the polymer concentration to 7 wt %. Alternatively, a much larger spacing of reactive groups along the polymer backbone or a more persistent polymer chain would favor intermolecular cross-linking—as we have previously demonstrated using oxidized alginate as the macromer reacting with **ADH**.^32^

To address the dramatic drop in hydrogel stiffness using 4-arm hydrazides compared to 2-arm hydrazides, we consider again the potential for intra-versus intermolecular cross-linking. As reactions between **pSM-***co***-OMAm** and both the PEG hydrazides possess similar reaction rates and similar chain properties, and we fix the concentration of reactive groups, the most prominent difference resides in the branched versus linear PEG chains and the resulting difference in the total mass content. In solution, 4-arm hydrazides possess a shorter mean-square radius of gyration (*R*_g_) compared to the 2-arm hydrazides. Thus, the average distance between the reactive monomers and the center of mass is shorter. With both a smaller *R*_g_ and higher valency (4-arm vs 2-arm), it follows that the average distance between reactive termini is shorter. Consequently, 4-arm hydrazides possess a higher effective local concentration of connected hydrazides, which is expected to favor a higher degree of intramolecular cross-linking compared to 2-arm PEGs. To experimentally probe whether a significant difference in intra- versus intermolecular cross-linking is likely, we performed DLS measurements on dilute solutions of **pSM-***co***-OMAm** reacted with either **20K-b** or **20K-t** (Figure S6). This study revealed that under dilute conditions, the addition of the tetrafunctional cross-linker (**20K-t**) led to a slight decrease in the apparent particle size, whereas the bifunctional cross-linker produced several populations of larger sizes, supporting the idea of increased intramolecular cross-linking with a tetrafunctional cross-linker. While encouraging, future studies across a broader range of molecular weights, valencies, and concentration regimes will be needed to quantitatively describe (and subsequently leverage) this behavior.

An intramolecular cross-link acts as a loop defect and reduces the effective functionality of 4-arm cross-linkers to three or two elastically active branches at rest—loop entanglements can contribute to out-of-equilibrium behavior. Controlling the concentration of primary loop defects has been used by Appel and colleagues to reduce *G*′ by up to an order of magnitude in ideal telechelic PEG networks.^68^ Additionally, at a constant total hydrazide function concentration, there will be twice as many 2-arm cross-linker molecules as 4-arm. The lower concentration of 4-arm cross-linking molecules will reduce the number of physical entanglements and overall concentration of elastically active cross-links. In turn, the network will be more sensitive to defects (loops, dangling chain ends, etc.) as their relative impact will be larger at low junction concentrations. Consequently, the lower concentration of cross-linking molecules and overall concentration of elastically active junctions may explain the much larger drop in stiffness observed with **20K-t** and **5K-t**.

Following the same reasoning, a dominance of intramolecular cross-linking could retard the formation of a contiguous network (gelation), which may explain the delay in the onset of cross-linking in both 4-arm formulations. However, by this same logic alone, we would also expect a further delay in the time needed for the network to reach thermodynamic equilibrium (*G*_P_), which is the opposite of what we observe. This may be due to the kinetic trapping of polymer chains, where the high functionality of 4-arm cross-linkers and **pSM-***co***-OMAm** requires a prohibitive number of simultaneous unbinding events for reorganization toward thermodynamic equilibrium to occur on the timescales we measured.^67^

Finally, we examined the surprising impact that a higher cross-linker valency had on the critical strain and stiffening-index of our hydrogels. Evidence is emerging that strain-stiffening in dynamic covalent networks does not follow traditional mechanisms.^60^ Webber and co-workers recently proposed a hybrid model to explain their observed strain stiffening behavior in ideal dynamic covalent boronic acid ester hydrogels, arguing that semiflexible ideal PEG networks stiffening arises from a combination of both entropic (chain elongation) and enthalpic (bond strain) elasticity.^60^ We refer the reader to their discussion for a detailed explanation. A salient proposition of their work is that a decrease in cross-link density (and stiffness) allows a greater degree of individual chain stretching and thus increased strain-stiffening (σ_c_, *m*) in dynamic covalent networks. This proposed mechanism aligns with our hypothesis that the higher valency **20K-t** and **5K-t** favor intramolecular cross-linking, reducing the effective cross-link density (and stiffness) of our hydrogels.

The data acquired during this investigation have highlighted how, despite the prevalent use and exciting developments shown using dynamic covalent networks, fundamental discrepancies remain that we have yet to fully elucidate and quantify. We aim to use dynamic systems, including the PEG hydrazide cross-linked **pSM-***co***-OMAm** presented here, to probe this emergent behavior systematically in the future. We anticipate that a deeper fundamental understanding of the relationship between dynamic covalent network junctions and macroscopic mechanical behavior will enable the robust engineering of soft dynamic networks with targeted mechanical properties.

## 4 Conclusions

Here, we systematically determined the RECs for a series of mono- and bivalent hydrazides of differing lengths when reacting with either a small molecule or polymeric aliphatic aldehyde. Our results demonstrated that the reactivity of terminal hydrazides is largely unaffected by cross-linker length in studies with a model small-molecule aldehyde. Furthermore, our RECs for a small-molecule aliphatic aldehyde reacting with an aliphatic aldehyde macromer were comparable to those presented in similar small-molecule model systems. However, a system comprising two macromers (of which one is telechelic) exhibited reduced apparent reactivity (≈10-fold lower *k*_1_ and resulting *K*_eq_) compared to a mixed system containing a small-molecule cross-linker. We attributed this observation to the excluded volume effects of the two macromers.

When comparing how differences in cross-linker length and reactivity translated to hydrogel formation, we observed that longer bi- or tetravalent chains are required to cross-link a hydrogel when the macromer backbone is densely populated with reactive groups (29% aldehyde containing units in this case). The bivalent small-molecule **ADH** was unable to form a hydrogel up to a higher concentration (7 wt %). Comparing the bivalent hydrazides, the longer 20K chains led to slightly softer hydrogels. Their difference in *G*′ broadly followed the expected changes based on the proportionality between the plateau shear modulus and entanglement molecular weight in an affine network. However, the same trend did not hold true comparing tetravalent cross-linkers to their bivalent counterparts. These observations allow us to identify the propensity of higher valency cross-linkers to create hydrogels with lower shear moduli and bring into focus the competition of intra-vs intermolecular cross-linking as an avenue of future exploration in dynamic networks.

Our hydrogels also demonstrated strain-stiffening behavior, which is rare in synthetic materials, not easily accessible in purely covalent systems, yet advantageous for diverse applications, including biomimetic systems. Importantly, we were also able to lower the critical stress and increase the stiffening index by switching from a bivalent to tetravalent dynamic cross-linker.

These results highlight the impact of chain length on hydrazide reactivity, with important differences in reactivity occurring when the reaction occurs between two macromolecules. This observation is important as these are the types of macromers commonly employed in hydrogel systems in the literature. Furthermore, in the context of rational dynamic hydrogel design, cross-linker valency also plays an important (yet so far underutilized) role in hydrogel mechanics. Simply by switching from a bivalent to tetravalent cross-linker, we were able to shift the strain-stiffening regime toward biologically relevant magnitudes. Whether this is due to network topology or stiffness and whether these trends underlie a fundamental tuning mechanism remains to be elucidated, and future work exploring a larger variety of cross-linker lengths and valencies will be needed to uncover quantitative descriptions. This information will be of use for translating existing kinetic studies on model dynamic systems to practical macromer hydrogel systems and in choosing or designing specific cross-linkers for a desired mechanical regime.

## Data Availability

The data that support the findings of this study are openly available in DataverseNL at 10.34894/KD1E7D.

## Supporting Information Available

Raw U-Vis kinetic traces, subsequent fitting of slices at the detection wavelength, stock solution preparation details, complete rheological data (time, frequency, and strain sweeps, with obtained strain-stiffening parameters), and DLS measurements (PDF)
