# Supplementary material for: Designing Dynamic Hydrogels: The Interplay of Cross-Linker Length, Valency, and Reaction Kinetics in Hydrazone-Based Networks
Source: Chem Mater. 2025 Apr 2;37(8):2709–19. doi: 10.1021/acs.chemmater.4c02573 (PMC12020002; doi:10.1021/acs.chemmater.4c02573)
Supplement: Supplementary file 1 — cm4c02573_si_001.pdf [file cm4c02573_si_001.pdf]

Supporting information

Designing Dynamic Hydrogels: The Interplay of Cross-Linker Length, Valency, and Reaction Kinetics in Hydrazone-based networks

Francis L. C. Morgan[1,2], Ivo A. O. Beeren[1,2], Lorenzo Moroni[2*], Matthew B. Baker[1,2*]

[1]Department of Instructive Biomaterials Engineering, MERLN Institute for Technology-Inspired Regenerative Medicine, Maastricht University, 6229 ER, Maastricht, The Netherlands

[2]Department of Complex Tissue Regeneration, MERLN Institute for Technology-Inspired Regenerative Medicine, Maastricht University, 6229 ER, Maastricht, The Netherlands

*Corresponding email: l.moroni@maastrichtuniversity.nl, m.baker@maastrichtuniversity.nl

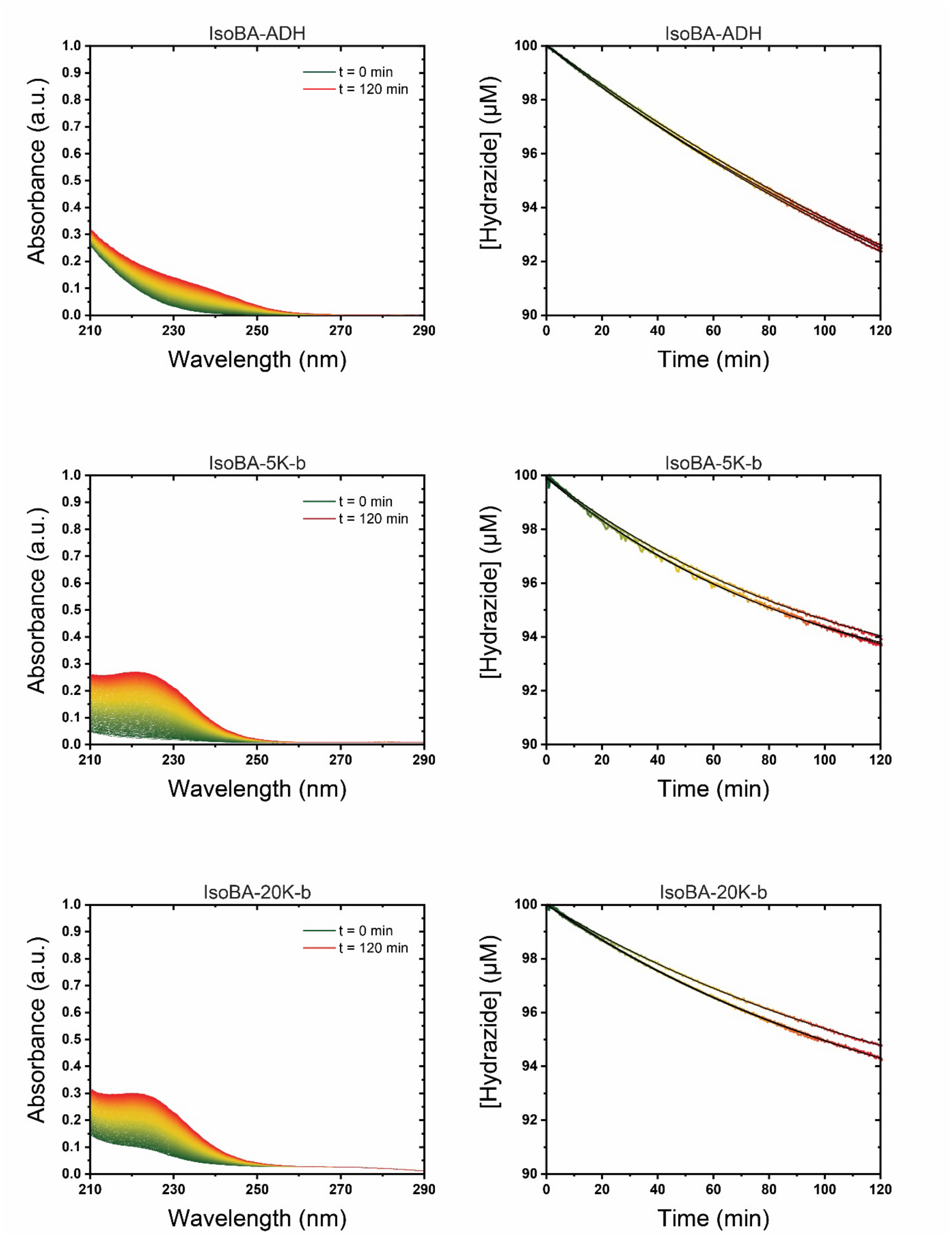

Figure S1. Raw and processed kinetic traces for each hydrazide+IsoBA pair investigated in this study. (Left) Raw kinetic traces for each pair taken over 2 h in PBS at pH 7.4 and 20 °C. (Right) Fitted curves of hydrazide consumption after processing. Each pair was measured in triplicate (*n* = 3).

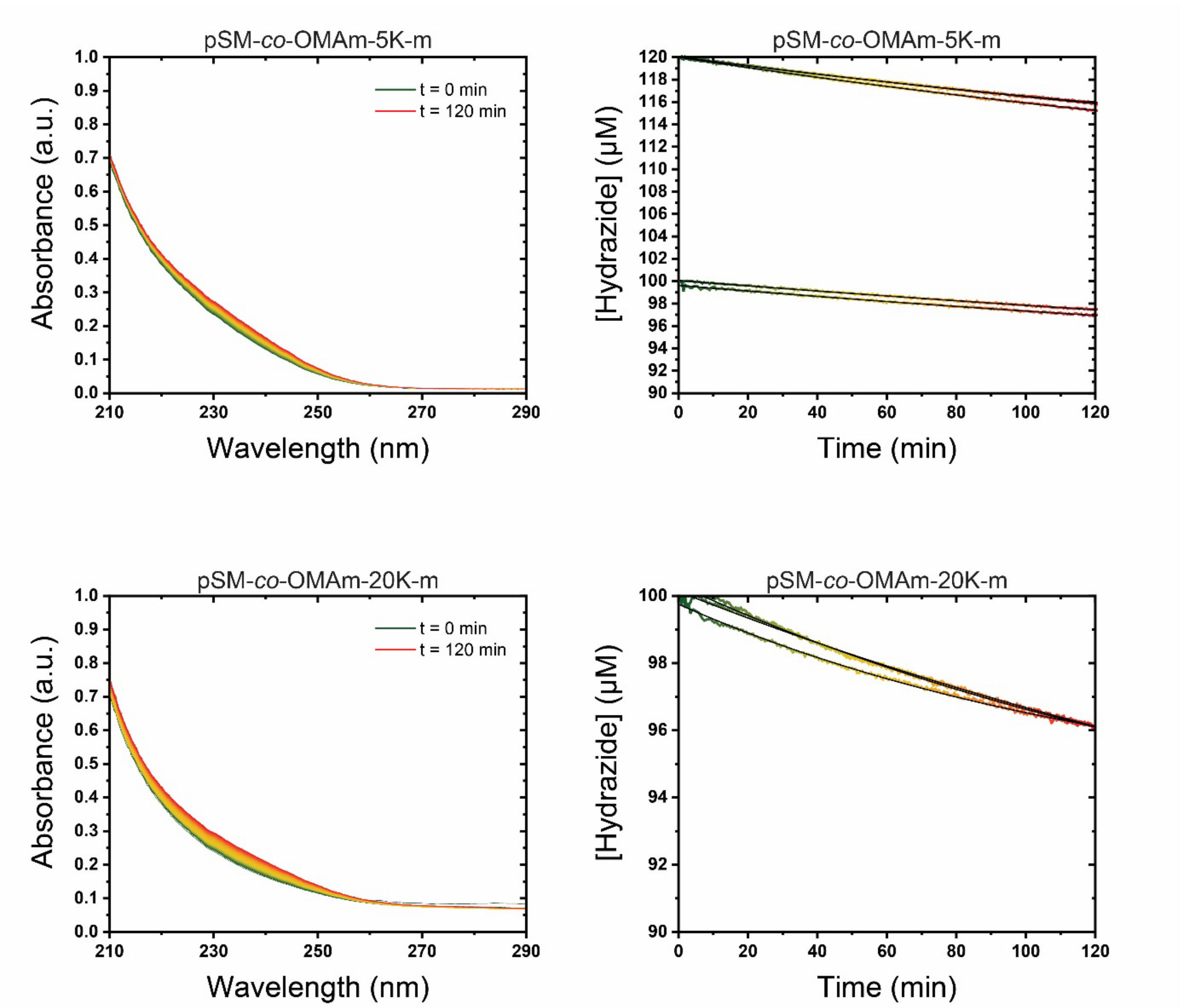

Figure S2. Raw and processed kinetic traces for each hydrazide+pSM-*co*-OMAm pair investigated in this study. (Left) Example of raw kinetic traces for each pair taken over 2 h in PBS at pH 7.4 and 20 °C. (Right) Fitted curves of hydrazide consumption after processing (n = 3–5). The different concentrations for **pSM-*co*-OMAm+5K-m** are due to a slightly difference volume of PBS used in 2 of the 5 replicates; no meaningful difference in the constants obtained from fitting was observed.

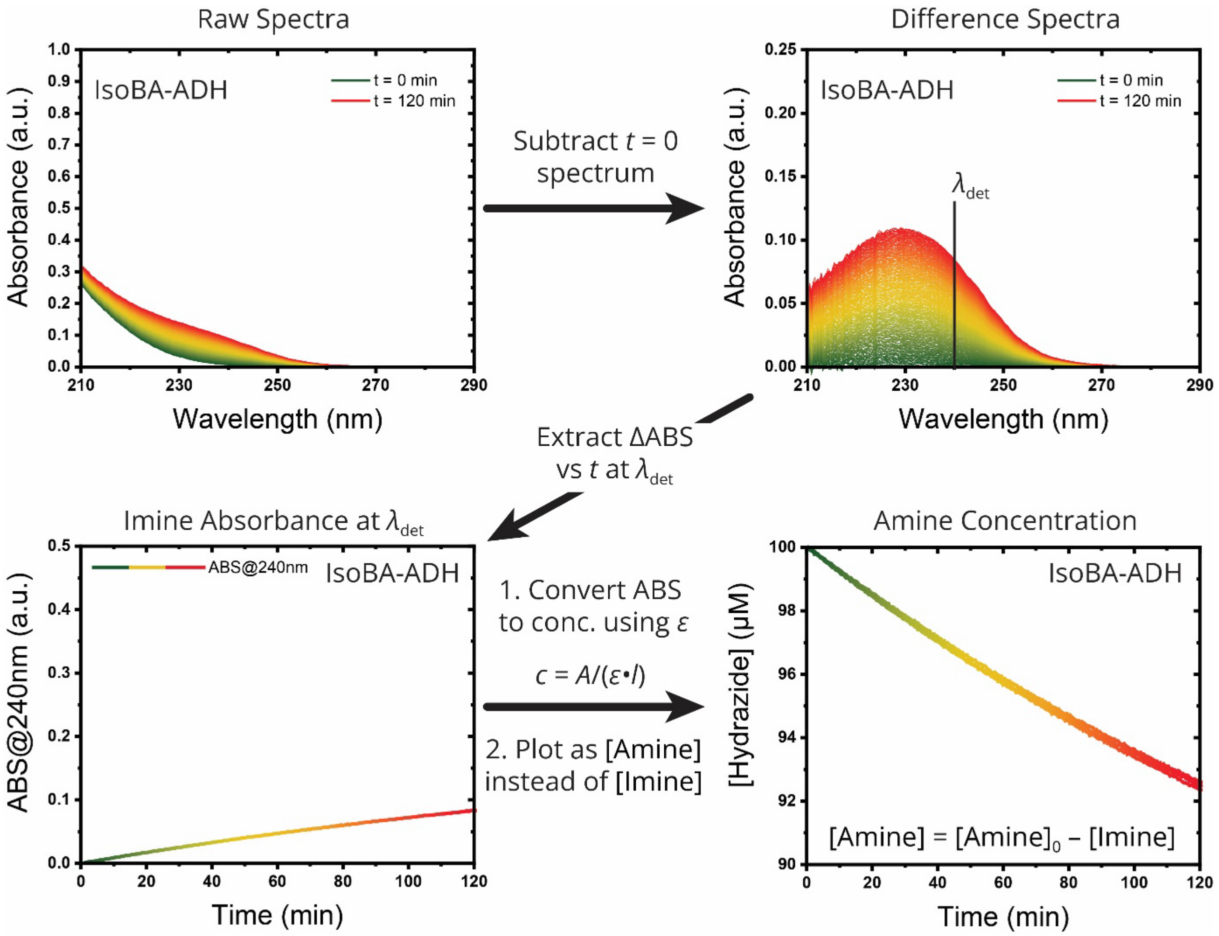

Figure S3. Processing of kinetic data prior to fitting. Once the raw data (top left) has been acquired, the initial scan at $t = 0$ is first subtracted to give the change in absorbance over time (difference spectra, top right). We then extract the change in absorbance over time due to imine formation (bottom left) at the specified detection wavelength. Finally, the imine absorbance value is converted to an amine concentration (bottom right) using the Beer-Lambert relationship alongside the known initial amine concentration. An amine concentration is required as the fitting model employed in this work is derived for amine consumption and not imine formation. The detection wavelengths (λ_{det}) and molar absorptivities (ε) were chosen based on our previous work while the fitting model employed was derived by Dirksen *et al*. See main text methods section (*Fitting of UV-Vis data to a second order bimolecular reversible rate equation to obtain rate and equilibrium constants*) for the relevant references.

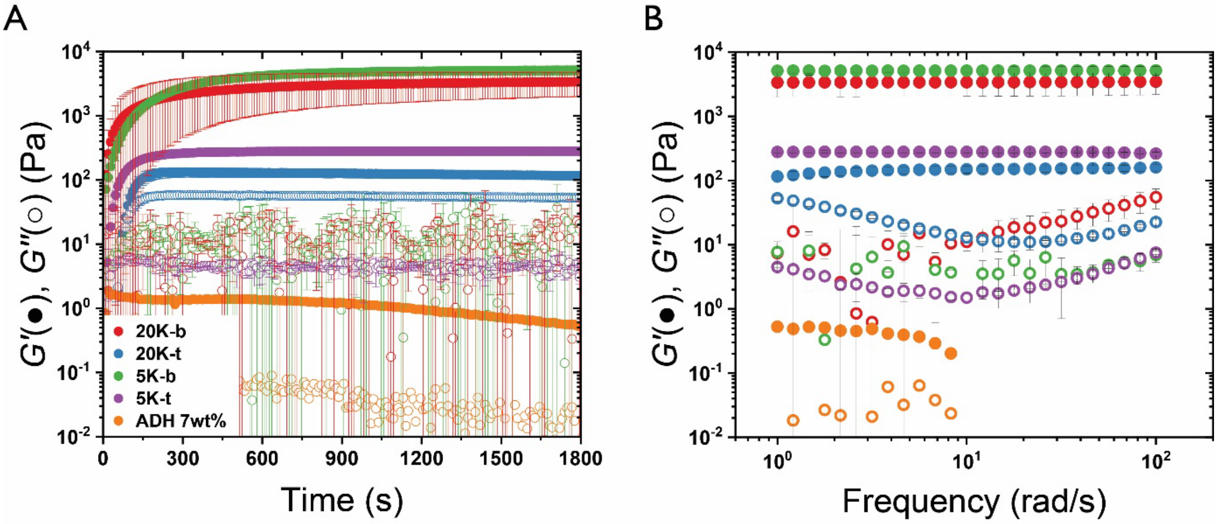

Figure S4. Time and frequency sweep data for the rheological study of bi- and tetra-valent PEG hydrazides forming gels with pSM-*co*-OMAm copolymer at a constant equimolar aldehyde and hydrazide concentration in PBS, at pH = 7.4 and 20 °C. (A) Time sweeps (γ = 1%, ω = 1 rad·s^{-1}, t = 1800 s, T = 20 °C) performed to monitor G' (closed) and G'' (open) during cross-linking. (B) Frequency sweeps (γ = 1%, ω = 1–100 rad·s^{-1}, T = 20 °C). The frequency sweep for ADH 7wt% is truncated to 10 rad·s^{-1} as inertial effects began to dominate – indicated by a sharp increase of the phase angel to > 90°. Values reported as the mean ± standard deviation of n = 3 replicates, with the exception of **20K-b**, which is n = 2.

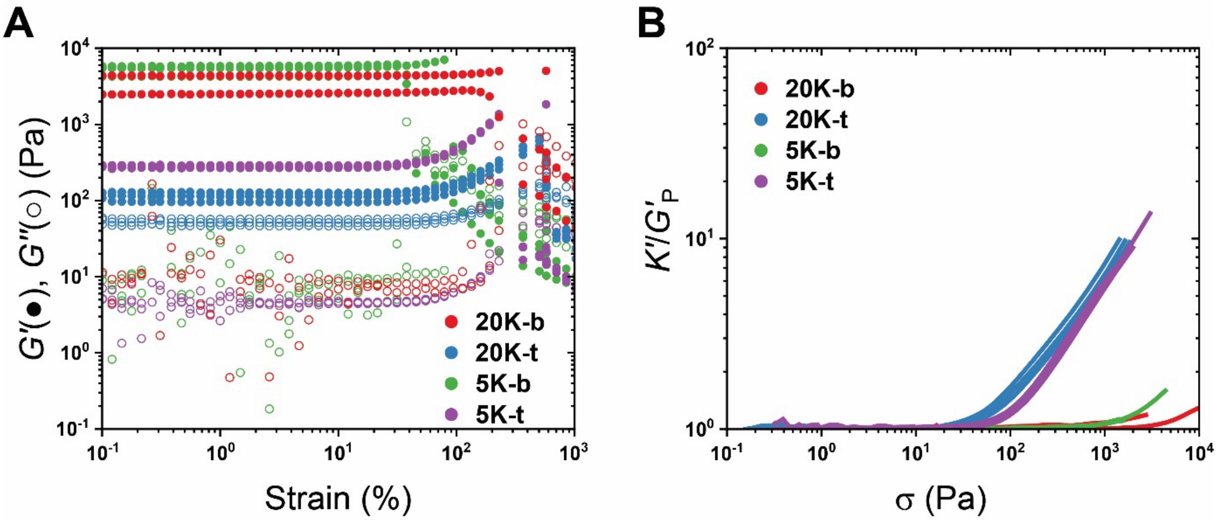

Figure S5. Strain sweep and differential modulus data for the rheological study of bi- and tetra-valent PEG hydrazides forming gels with pSM-*co*-OMAm copolymer at a constant equimolar aldehyde and hydrazide concentration in PBS, at pH = 7.4 and 20 °C. (A) Strain sweeps (γ = 0.1–1000%, ω = 1 rad·s^{-1}, T = 20 °C) showing G' (closed) and G'' (open), performed after cross-linking. (B) Differential modulus ($K' = \partial\sigma/\partial\gamma$) of all replicates, highlighting the strain-stiffening behavior of these formulations. Values reported as the mean ± standard deviation of n = 3 replicates, with the exception of **20K-b**, which is n = 2.

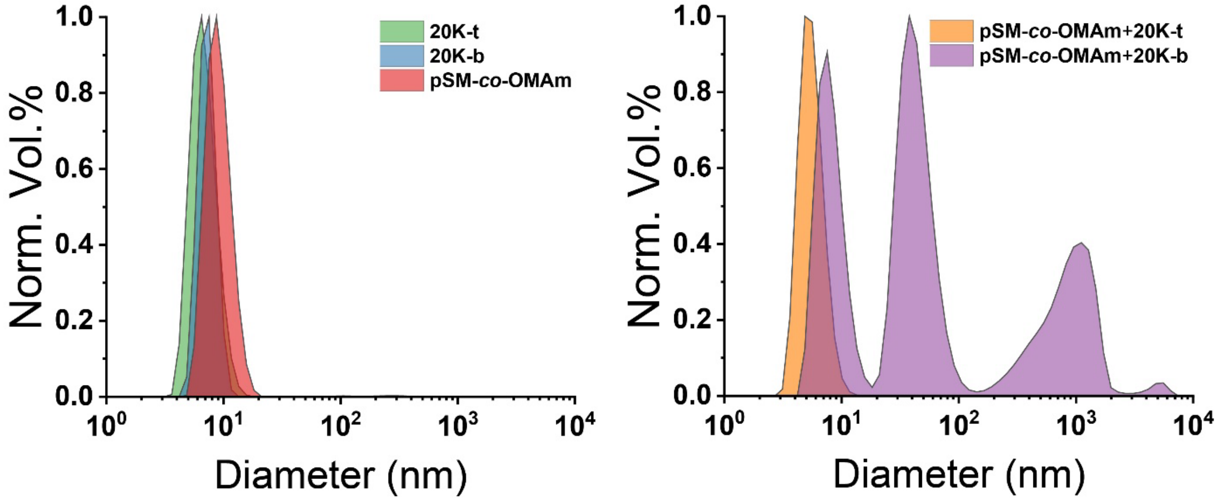

Figure S6. Dynamic Light Scattering (DLS) particle size distribution using a bi- vs tetra-functional hydrazide cross-linker. (Left) Normalized volume distribution for the copolymer with pendant aldehydes (**pSM-*co*-OMAm**; M_w = 36.4 kg·mol^{-1}), and 20K PEG hydrazide cross-linkers that are bi- (**20K-b**) or tetra-functional (**20K-t**). Similar molecular weights give rise to similar initial apparent particle sizes. (Right) Upon reacting **pSM-*co*-OMAm** with **20K-b** under dilute conditions we observe a distribution of several large particle populations. In contrast, reacting **pSM-*co*-OMAm** with **20K-t** reveals a single population of even smaller particles. Note that these values should only be considered relative to one another and not taken as absolute, since they rely upon the assumption of spherical particles with known absorbance and refractive index, which have not been verified.

Table S1. Preparation of stock solutions for hydrogel preparation.

Stock compound	M_w ($g{\cdot}mol^{-1}$)	Mass (mg)	[Hyd][a] (mM)	V (µL)	Purity	ρ[b] ($g{\cdot}cm^{-3}$)	Solids (wt%)
**pSM-*co*-OMAm**[c]	223.97[d]	39.0	49.3	1025	1.0	1.05	3.81
	223.97[d]	12.3	185.2	86	1.0	1.05	14.30
**ADH**[c]	174.2	4.4	50.0	1000	0.98	1	0.44
	174.2	26.2	200.0	1474	0.98	1	1.78
**5K-b**	5000	26.3	47.6	210	0.95	1.05	12.53
**20K-b**	20000	94.40	52.0	172.5	0.95	1.15	54.72
**5K-t**	5000	12.4	44.9	210	0.95	1.05	5.90
**20K-t**	20000	53.6	44.3	230	0.95	1.15	23.30

[a] [Hyd] refers to the concentration of hydrazide functions (not molecules) in the final volume after adjusting for the density.

[b] The density of these solutions was estimated by measuring the new volume using a micropipette after dissolution.

[c] A second, more concentration stock solution was prepared for the preparation of the 7 wt% **pSM-*co*-OMAm** formulation containing 1 equiv **ADH**.

[d] The given molecular weight is the average monomer molecular weight, of which 29% are aldehyde containing monomer units.

Table S2. Critical stress (σ_c) and stiffening index (m) for hydrazide hydrogels using difference cross-linker length and valency.

Formulation	**pSM-co-OMAm** (wt%)	Total (wt%)	σ_c[a] (Pa)	m
**20K-b**	1.5	21.3	1930 ± 1960	0.16 ± 0.09
**20K-t**	1.5	11.4	75 ± 10	0.73 ± 0.01
**5K-b**	1.5	6.5	750 ± 440	0.18 ± 0.15
**5K-t**	1.5	4.0	100 ± 6	0.75 ± 0.02

[a] The high error of bi-valent hydrazides results from variation in the strain at break among replicates. The method employed to calculate σ_c takes the final 5 data points prior to rupture, shifting the slope significantly when breaking at different points.
